# Single-Cell Transcriptomics Reveals Core Regulatory Programs That Determine the Heterogeneity of Circulating and Tissue-Resident Memory CD8^+^ T Cells

**DOI:** 10.3390/cells10082143

**Published:** 2021-08-20

**Authors:** Yao Chen, Jian Shen, Moujtaba Y. Kasmani, Paytsar Topchyan, Weiguo Cui

**Affiliations:** 1 Versiti Blood Research Institute, Milwaukee, WI 53213, USA; yaochen.immunity@gmail.com (Y.C.); jshen@mcw.edu (J.S.); mkasmani@mcw.edu (M.Y.K.); ptopchyan@mcw.edu (P.T.); 2 Department of Microbiology and Immunology, Medical College of Wisconsin, Milwaukee, WI 53226, USA

**Keywords:** CD8 tissue-resident memory T cell, LCMV infection, single-cell RNA-sequencing, heterogeneity, transcriptional regulation, transcription factors, GP33

## Abstract

During acute infections, CD8^+^ T cells form various memory subpopulations to provide long-lasting protection against reinfection. T central memory (TCM), T effector memory (TEM), and long-lived effector (LLE) cells are circulating memory populations with distinct plasticity, migration patterns, and effector functions. Tissue-resident memory (TRM) cells permanently reside in the frontline sites of pathogen entry and provide tissue-specific protection upon reinfection. Here, using single-cell RNA-sequencing (scRNA-seq) and bulk RNA-seq, we examined the different and shared transcriptomes and regulators of TRM cells with other circulating memory populations. Furthermore, we identified heterogeneity within the TRM pool from small intestine and novel transcriptional regulators that may control the phenotypic and functional heterogeneity of TRM cells during acute infection. Our findings provide a resource for future studies to identify novel pathways for enhancing vaccination and immunotherapeutic approaches.

## 1. Introduction

During acute infection, antigen-specific CD8^+^ T cells are activated and differentiate into cytotoxic effector cells to clear pathogens. Most of these effector cells die by apoptosis after resolution of the primary infection. However, some survive and form memory cells, which provide long-lasting protection against previously encountered infections. Based on the migration patterns and functions, heterogeneous populations of memory CD8^+^ T cells have been identified in blood, secondary lymphoid organs (SLOs), and peripheral tissues. T central memory (TCM) cells exhibit the highest plasticity, which allows them to expand and differentiate into effector cells upon reinfection [1]. TCM cells have high expression of CD62L and CCR7 which are SLOs homing molecules [1]. T effector memory (TEM) cells lack these homing molecules and provide immediate pathogen control via higher effector functions [1]. Recently, studies have revealed additional heterogeneity within the classically defined TEM population, including peripheral memory (TPM) cells, which exhibit an intermediate level of CX_3_CR1 and patrol between peripheral tissues and blood [2]. Furthermore, recently identified long-lived effector (LLE) cells, which are distinguished by the highest level of CX_3_CR1 among TEM-like cells have the most robust cytotoxic function when compared with other memory CD8^+^ T cells [3]. LLE cells share similar features with CX_3_CR1^hi^ TEM and further studies may be needed to determine if these two populations are, in fact, identical. In addition to these circulating memory subsets, tissue-resident memory (TRM) cells are found in various tissues, especially at barrier surfaces, such as the skin, lung, female reproductive tract, and intestinal epithelium [4,5]. They express tissue residency molecules, such as CD103 and CD69, and maintain the ability to produce effector molecules such as granzyme B (GzmB), TNF, IFN-γ, and IL-2 depending on their site of residence [5]. Whereas heterogeneity within circulating memory CD8^+^ T cells has been well-characterized, TRM cell heterogeneity has only been investigated relatively recently and requires further investigation.

The role of transcription factors (TFs) in regulating gene expression and subset differentiation of memory cells has received considerable attention. For example, TFs including Id2 [6], T-bet [7], and Blimp1 [8] are required for TEM formation. Id3 [9], Eomes [10], Bcl6 [11], Foxo1 [12], and Tcf-1 [13,14] are critical for TCM differentiation. The TFs that control the newly identified memory subsets, such as LLE cells, are currently unknown. Furthermore, although Runx3 [15], Notch [16], Bhlhe40 [17], and Blimp1 and its homolog Hobit [18] are reported to regulate TRM formation, it remains unclear whether potentially heterogeneous subpopulations of TRM cells may be controlled by distinct TFs.

Here, we applied single-cell RNA sequencing (scRNA-seq) on GP33^+^ CD8^+^ T cells from spleen and small intestine post LCMV-Armstrong (LCMV-ARM) infection to test the heterogeneity of circulating memory populations and TRM cells. Furthermore, we used Single-Cell rEgulatory Network Inference and Clustering (SCENIC) analysis on our scRNA-seq data to examine the gene regulatory networks (GRNs) that contribute to the distinct transcriptomic profiles and functions of the various memory subsets. In doing so, our study provides a framework to systematically identify critical regulators for each CD8^+^ memory T-cell population.

## 2. Materials and Methods

### 2.1. Mice and LCMV Infection

Six- to eight-week-old female C57BL/6 were purchased from Charles River. Mouse handling conformed to the requirements of the Institutional Animal Care and Use Committee (IACUC) guidelines of the Medical College of Wisconsin (MCW).

Mice were infected with 2 × 10^5^ PFU/mouse LCMV-ARM by intraperitoneal injection to establish acute infection. LCMV-ARM was prepared by a single passage on BHK21 cells and viral titers were determined by plaque assay on Vero cells.

### 2.2. Isolation of Lymphocytes from Spleen and Small Intestines

For isolation of lymphocytes from the spleen, spleens were mashed and passed through 70-µm cell strainers to form single-cell suspension. Then, ACK Lysing Buffer (Thermo Fisher Scientific, Waltham, MA, USA) was used to remove red blood cells. For isolation of intraepithelial lymphocytes (IELs) from small intestines, small intestines were collected from stomach to caecum. Then, they were cut open longitudinally and washed with cold PBS with 2% FBS. Small intestines were cut into pieces and incubated in RPMI medium with 10% FBS and 5 mM EDTA for 30 min at 37 °C while shaking. IELs were suspended in the supernatant after vortexing the tube containing the small intestines and were further enriched by using a 44%/56% Percoll density gradient. Cells were centrifuged at 2000 rpm for 20 min without applying brakes.

### 2.3. Cell Sorting

All flow cytometry data were acquired on an LSRII (BD Biosciences) flow cytometer with BD FacsDiva software (Version 8) and analyzed by FlowJo (Version 9). A BD FACSAria cell sorter was used for sorting. Cells were stained with GP33 tetramer (1:100, provided by the NIH tetramer core facility) and the following antibodies were used against cell surface antigens for 30 min at 4 °C: anti-CD8α (clone 53–6.7, 1:200), anti-CD44 (clone 1 M7, 1:200), anti-CD103 (clone 2E7, 1:200), anti-CD62L (clone MEL-14, 1:200).

### 2.4. Single-Cell RNA Sequencing and Analysis

GP33^+^CD44^+^CD8^+^ cells were FACS-sorted from LCMV-ARM-infected mice on day 30 post-infection and loaded on the chromium controller (10× Genomics). scRNA-seq libraries were prepared using the Chromium Single Cell 5′ v1 Reagent Kit (10× Genomics) according to the manufacturer’s protocol. Libraries were loaded onto an Illumina NextSeq sequencing system with the NextSeq 500/550 High Output Kit v2 (150 cycles) (Illumina) with the following conditions: 26 cycles for read 1, 91 cycles for read 2, and 8 cycles for i7 index. Raw sequencing data were de-multiplexed and converted to gene-barcode matrices using the Cell Ranger (Version 2.2.0) mkfastq and count functions, respectively (10× Genomics). The mouse reference genome mm10 was used for alignment. Data were further analyzed in R (Version 3.4.0) using Seurat (Version 3). The number of genes detected per cell, number of unique molecular identifiers (UMIs), and the percent mitochondrial genes were plotted; outliers were removed (cells that expressed less than 200 and more than 2500 genes, and cells with >0.05 percent mitochondrial genes) to filter out doublets and dead cells. Differences in the number of UMIs and percent mitochondrial reads were regressed out. Raw UMI counts were normalized and log-transformed. We removed contaminating cells and kept α/β CD8^+^ T cells based on high expression of *Cd3e*, *Cd8a*, *Trac*, *Trbc1*, and *Trbc2.* To correlate our findings with previously reported TRM subsets, published scRNA-seq data on activated P14 T cells (CD8^+^ CD44^+^) cells from siIEL (GSE131847) [19,20] were used to perform intergraded analysis using Seurat.

### 2.5. SCENIC Analysis

Log-normalized UMI counts were used as the input gene expression values for SCENIC analysis [21]. First, the potential target genes of each TF were determined based on co-expression patterns using GENIE3 [22]. A threshold of 0.03 for Pearson correlation was used. Then, TF-motif enrichment analysis was performed to identify the regulons (TFs and their direct-binding targets) using RcisTarget [21]. Motifs with a normalized enrichment score (NES) >3.0 were kept. The search space was 10 kb around the TSS or 500 bp upstream of the TSS from the mm10 mouse reference genome. Finally, all genes in each cell were ranked by their expression and a curve was generated with the number of recovered genes from the regulon across the ranking of genes. AUCell [21] was then used to calculate the activity of each regulon as the area under the recovery curve (AUC) in each cell. AUCell thresholds for some regulons were manually adjusted as recommended by the SCENIC developers. Regulons that were active in at least 1% of cells and correlated (absolute Pearson correlation >0.30) with at least one other regulon were kept. The TF–gene interactions in the GRNs were generated based on the TF-motif enrichment analysis during SCENIC analysis.

### 2.6. Bulk RNA-Sequencing and Analysis

Three replicates of each cell population were sequenced with a modified SMART-Seq2 protocol [23]. Briefly, 1000 cells of each sample were directly sorted into 6.5 μL of cell lysis buffer consisting of 1 μL 2% Triton-X100 (Sigma-Aldrich, St. Louis, MO, USA), 0.5 μL RNase inhibitor (Takara, Shiga, Japan), 2 μL 10 μM oligo-dT30VN primer (IDT, Newark, NJ, USA), and 3 μL 10mM dNTP mix (ThermoFisher, Coralville, IA, USA). Sorted cells were immediately frozen on dry ice and stored in −80 °C. For reverse transcription, 4 μL 5× superscript II First-Strand Buffer (Invitrogen, Waltham, MA, USA), 1 μL 100 mM DTT, 0.5 μL RNase inhibitor, 0.2 μL 100 μM TSO (IDT), 4 μL 5 M Betaine (Sigma-Aldrich), 0.12 μL 1 M MgCl2 (Sigma-Aldrich) and 2 μL SuperScript II reverse transcriptase (Invitrogen) were added into the thawed cell lysate. After three minutes of incubation at 72 °C, samples were put back on ice and incubated with the following program: 42 °C × 45 min, 70 °C × 10 min, 4 °C hold. cDNA amplification and library preparation were done as described in the SMART-Seq2 protocol. Pooled libraries were sequenced with a NextSeq 500 sequencer using a high output V2.5 75 cycle kit (Illumina, San Diego, CA, USA) and 2 × 37 paired-end reads. Bulk RNA-seq data were aligned to mm10 and quantified with Salmon [24]. Differential analysis of gene expression among TCM, TEM, and TRM was done using DESeq2 (Version 1.30.1) [25]. Differently expressed genes (*p*.adjust < 0.05) in each pair-wise comparison were used for making the heatmap. Gene set enrichment analysis (GSEA) was done with the R package *clusterProfiler* using pathways from the KEGG database [26].

## 3. Results

### 3.1. Single-Cell Transcriptomics Probes Heterogeneity within Memory CD8^+^ T-Cell Populations

**Figure 1:**
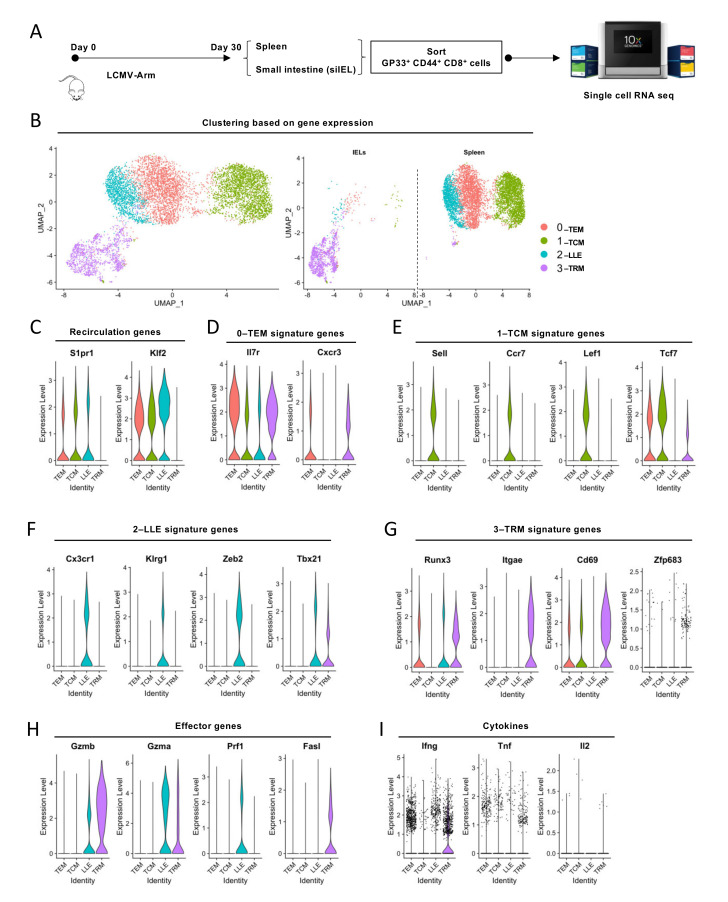
Single-cell transcriptomics probes heterogeneity within memory CD8^+^ T-cell populations: (**A**) Schematic of experimental set-up. Mice were infected with 2 × 10^5^ PFU/mouse LCMV Armstrong. On day 30 post-infection, GP33^+^CD44^+^CD8^+^ cells were FACS-sorted from spleen and siIEL. scRNA-seq libraries were generated using the 10× Genomics platform. (**B**) Unsupervised clustering based on gene expression identified four major populations when visualized by UMAP. Left, combined scRNA-seq datasets from spleen and siIEL. Right, cells from different tissues. (**C**–**I**) Violin plots showing the expression of signature genes of different memory subsets and genes related to migration, effector function, and cytokines, as well as signature genes of the three major memory T-cell subsets.

First, we performed scRNA-seq on GP33^+^ CD44^+^ CD8^+^ cells from spleen lymphocytes and siIELs 30 days post-LCMV ARM infection (Figure 1A and Supplementary Figure S1A). About 90% of GP33^+^ CD44^+^ CD8^+^ cells from siIELs expressed CD103, which is the predictive marker of intraepithelial CD8^+^ T cells (Supplementary Figure S1A). Then, these two scRNA-seq datasets were integrated using the Seurat package and underwent unsupervised Uniform Manifold Approximation and Projection (UMAP) analysis. Our analysis identified four transcriptionally distinct clusters (Figure 1B). Clusters 0–2 but not Cluster 3 exhibited *Klf2* and *S1pr1* expression, which mediate egress of T cells into the circulation [27] (Figure 1C). We also observed that Clusters 0–2 expressed genes previously associated with TEM (*Il7r*, *Cxcr3*, intermediate levels of *Cx3cr1* and *Klrg1*), TCM (*Sell*, *Ccr7*, *Lef1*, and *Tcf7*), and LLE (high levels of *Cx3cr1*, *Klrg1*, *Tbx21* (encodes T-bet), and *Zeb2*), respectively [3,19,20] (Figure 1D–F). CD62L and CCR7 are two key molecules required for LN homing [1]. TCF1 and LEF1 can be induced by WNT signaling and are essential for the stemness and quiescence of TCM cells [13,28]. CX_3_CR1 is a chemokine receptor essential for homing to the vasculature [29]. KLRG1 and ZEB2 promote terminally differentiated effector CD8^+^ T cells [7,30]. The expression level of CX_3_CR1, KLRG1, and T-bet are reported to correlate with the cytotoxicity of CD8^+^ T cells [2,7,31]. Cells from Cluster 3 almost exclusively came from the siIEL sample (Figure 1B) and expressed genes previously reported to be associated with TRM cells, including *Itgae* (encodes CD103), *Cd69*, *Zfp683* (encodes Hobit), and *Runx3* [32] (Figure 1G). CD103 and CD69 are the two most commonly used markers for TRM cells. CD103 mediates tissue residency through interaction with E-cadherin, expressed in epithelial and neuronal tissues [33]. CD69 regulates tissue retention by antagonizing S1P1-mediated tissue egress [34]. Hobit and RUNX3 are two transcription factors that construct the transcriptional program of tissue residency [15,18]. Furthermore, we found that both LLE and TRM cells had elevated expression of genes associated with cytotoxicity, such as *Gzma*, *Gzmb*, and *Ifng* (Figure 1H). TEM cells expressed *Tnf* and *Ifng* (Figure 1I). TCM cells exhibited the highest level of *Il2*, while LLE cells had the lowest (Figure 1I). These data revealed the heterogeneity of circulating CD8^+^ memory T cells and the unique transcriptional features of TRM cells.

### 3.2. Single-Cell Network Inference Reveals Candidate Regulators of Memory CD8^+^ T-Cell Populations

**Figure 2:**
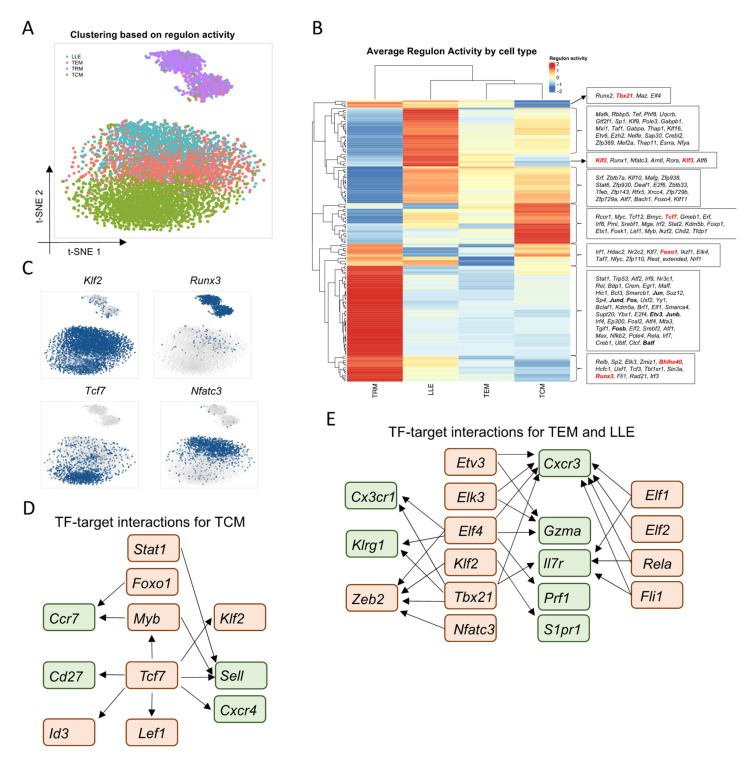
Single-cell network inference reveals candidate regulators of memory CD8^+^ T-cell populations: (**A**) Unsupervised clustering based on regulon activity separated cells into four major populations when visualized by t-SNE. Colors denote the CD8^+^ T-cell subsets identified by gene expression profiles in Figure 1. (**B**) Heatmap showing the average regulon activity in each cell population. Scale bar denotes SCENIC AUC score. (**C**) t-SNE projections showing binary regulon activity of example regulons for different memory subsets. (**D**,**E**) Gene regulatory networks showing TF-target interactions for TEM, LLE, and TCM. Key TFs are highlighted in red, putative regulated genes are highlighted in green.

To comprehensively reconstruct the gene regulatory networks for different types of CD8^+^ T-memory subsets during acute viral infection, we applied network inference on our scRNA-seq data of LCMV-specific CD8^+^ T cells using SCENIC [21]. SCENIC predicts TFs alongside their candidate target genes, which are jointly called a regulon, via co-expression patterns and *cis*-regulatory motif analysis. Here, we identified 153 regulons that were active in at least 1% of the total cells from our scRNA-seq data. Then, the activity of these regulons was accessed in individual cells which were reclustered based on regulon activity patterns (Figure 2A). By then labeling each individual cell with its Seurat-derived cluster identity, we found that TRM cells harbor the most unique regulon activities while TEM, TCM, and LLE cells shared relatively similar regulon profiles (Figure 2A,B). Nevertheless, we found some regulon heterogeneity among the non-TRM populations, as expected. TCM cells lacked *Tbx21* regulon activity but were enriched for *Tcf7* regulon activity, which has been reported to regulate the stemness of TCM [28] (Figure 2B,C). Furthermore, we found that *Tcf7* might program TCM cells through the regulation of signature genes, including *Klf2*, *Id3*, and *Sell* (encodes CD62L) (Figure 2D). TEM and LLE cells both had high activity of regulons related to T-cell migration and activation, such as *Klf2*, *Klf3*, and *Nfatc3* (Figure 2B,C,E). We found that TFs, including *Etv3*, *Elk3*, and *Elf4*, may regulate *Gzma* and *Gzmb* expression to promote the cytotoxicity of LLE cells (Figure 2E). Furthermore, ELF4 has been shown to regulate the proliferation and migration of CD8^+^ T cells via KLF2 [35].

In line with previous research [32], TRM cells were characterized by enriched activity of *Runx3* and *Foxo1* regulons, as well as a lack of activity of *Klf2* and *Klf3* regulons (Figure 2B,C). In addition to previously reported TFs, we found that TRM cells have high activity of AP-1 TF family members, including *Jun* (encodes c-Jun), *Junb*, *Jund*, *Fos*, *Fosb*, and *Batf* (Figure 2B). Furthermore, TFs induced by interferon signaling, including *Stat1*, *Irf1*, *Irf7*, and *Irf9* were enriched in TRM cells (Figure 2B). TFs related to the NF-κB signaling pathway, such as *Bcl3*, *Rela*, *Relb*, *Rel*, and *Nfkb2*, were also enriched in TRM cells (Figure 2B). In addition, we also found TRM-enriched regulons that have previously been reported to regulate T-cell differentiation and function in broader settings, but whose roles in TRM cells specifically have not been determined. These included *Trp53* [36], *Nr3c1* [37], and *Fli1* [38]. In summary, we identified known and unknown TFs that may contribute to the phenotypic heterogeneity of memory CD8^+^ T cells during viral infection. Furthermore, TRM cells are not only transcriptionally distinct from circulating memory populations, but their differentiation and function are regulated by unique gene regulatory networks.

### 3.3. Bulk RNA-Seq Reveals the Unique Transcriptional Profiles of TRM Cells

**Figure 3:**
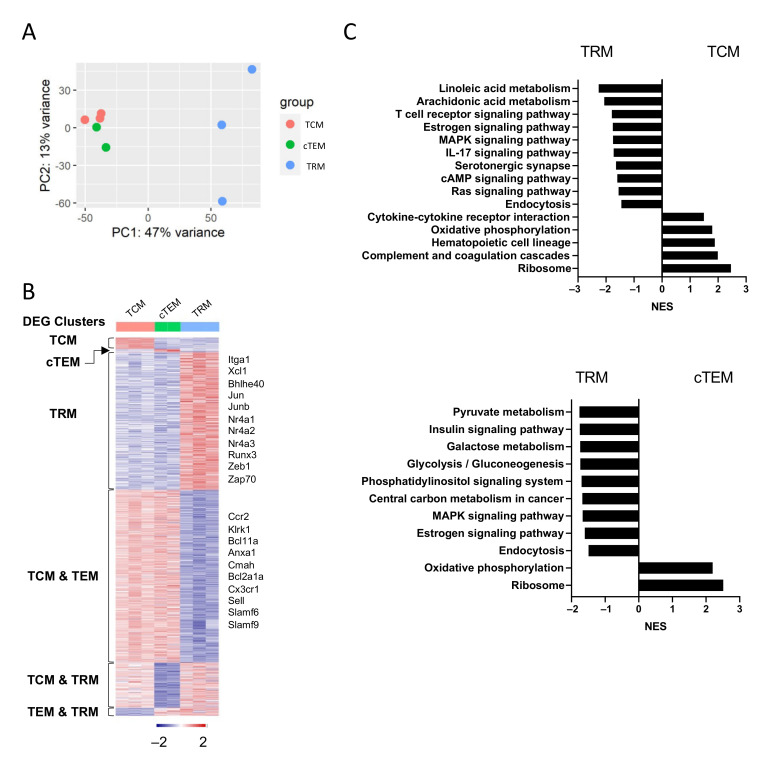
Bulk RNA-seq reveals the unique transcriptional profiles of TRM compared with TEM and TCM: (**A**) Principal component analysis (PCA) plot showing the top two principal components distinguishing the transcriptional profiles of three populations. (**B**) Heatmap showing the significantly (*p*.adjust < 0.05) differently expressed genes in each population. (**C**) Gene set enrichment analysis (GSEA) using the KEGG database revealed pathways up or downregulated (adjusted *p*-value < 0.05) in TRM compared with TCM or TEM.

To further compare the transcriptional profiles of TRM cells versus circulating memory CD8^+^ T-cell populations, we performed bulk RNA-sequencing (RNA-seq) with cells sorted at day 30 post-infection with LCMV-ARM. TRM cells (GP33^+^ CD44^+^ CD103^+^ CD8^+^) were isolated from siIEL in the same way as in Supplementary Figure S1A. The conventionally defined effector memory (cTEM) and TCM were sorted as CD62L^+^ GP33^+^ CD44^+^ CD8^+^ and CD62L^−^ GP33^+^ CD44^+^ CD8^+^ T cells from spleen, respectively. Based on principal component analysis (PCA), TCM and cTEM cells showed considerable similarity while TRM cells were more transcriptionally distinct from the other two populations (Figure 3A). To identify the gene sets uniquely expressed in or shared between each population, we performed differential analysis by comparing the three populations pairwise (TCM vs cTEM, TCM vs TRM, and cTEM vs TRM). The differentially expressed genes (DEGs) (adjusted *p*-value (padj) < 0.05, absolute log2(fold change) (log2(FC)) > 0.5) fell into 6 expression patterns: three clusters of DEGs uniquely upregulated in a single population (TCM, cTEM, or TRM) and three clusters of DEGs upregulated by two populations (TCM and cTEM, TCM and TRM, or cTEM and TRM) (Figure 3B). Consistent with the PCA results, TRMs had the most differentially expressed genes compared to TCM or cTEM (Figure 3B). In addition to *Cd69*, *Runx3*, and NR4A family members (*Nr4a1*, *Nr4a2*, and *Nr4a3*), TRM-associated genes identified by scRNA-seq (*Jun*, *Junb*, *Fos*, etc.) were also confirmed to be upregulated in TRMs by bulk-RNA seq. On the other hand, genes associated with TCM and TEM (*Sell*, *Cx3cr1*, etc., were found to be downregulated in TRM cells.

With the identified DEGs, we performed Gene Set Enrichment Analysis (GSEA) to further understand the pathways enriched in TRM cells (Figure 3C). Compared with TCM and cTEM, TRM cells upregulated the MAPK signaling pathway (Figure 3C). The enrichment of MAPK signaling pathways and upregulation of AP-1 family members (Figure 2B) might indicate a more activated status of TRM cells [39] possibly due to the stimuli from the tissue environment. Estrogen receptor α was reported to promote T-cell activation, proliferation, and cytokine production [40]. TRM cells were also found to be more active in fatty acid metabolism (Figure 3C), which has previously been reported to support their long-term survival and function [41,42].

Consistent with a recent study showing the CD62L^−^ CD8 subset can be further divided into LLE and TEM cells [3]. We identified three circulating memory subsets in our scRNA-seq experiment (Figure 1B). To compare TRM cells with the newly identified TEM and LLE cells, we utilized published bulk RNA-seq data of TEM (CD44^+^ CD62L^−^ KLRG1^−^ CD27^+^) and LLE (CD44^+^ CD62L^−^ KLRG1^+^ CD27^+^) cells from spleen (GSE152841) [3]. Differential expression analysis of our TRM cells versus the TEM or LLE cells revealed DEGs relatively upregulated or downregulated in TRM cells. Due to the similar transcriptomic profiles of TEM and LLE cells [3], the DEGs relative to TEM and LLE largely overlap with each other (Supplementary Figure S2A). For example, TRM cells expressed higher levels of genes related to tissue residency (*Cd69* and *Itgae*), cytotoxicity (*Gzmb* and *Gzmk*), lipid uptake and intracellular transport (*Fabp1* and *Fabp2*), and TCR signaling (*Zap70* and *Nr4a* TFs) (Supplementary Figure S2B). Furthermore, compared to TEM and LLE subsets, TRM cells downregulated genes related to migration, like *Cx3cr1*, *S1pr1*, and *S1pr5* (Supplementary Figure S2B). However, the bulk RNA-seq data of TEM and LLE cells were generated from splenic memory CD8 T-cell subsets 90 days post-LCMV-Arm infection [3] while we used TRM cells from day 30 post-infection. The phenotype of TRM cells might change over time [20].

### 3.4. Core Regulatory Programs That Determine Heterogeneous TRM Populations in siIELs

Recent studies have revealed heterogeneity within the TRM cell population [19,20]. This work has shown that there are two major subsets of TRM CD8^+^ T cells in response to LCMV-ARM infection in mice: effector-like Id3^lo^ Blimp1^hi^ TRM cells and memory-like Id3^hi^ Blimp1^lo^ TRM cells [19]. Here, we have also observed that TRM cells appear to form two clusters rather than a unified one based on their regulon activities (Figure 2A), indicating that distinct gene regulatory networks control the differentiation and function of TRM subsets. To correlate our findings with previously reported TRM subsets, we used scRNA-seq integration to identify cross-dataset populations that have a similar biological state by combining our data with previously published data (GSE131847) [19,20] (Supplementary Figure S3A). Consistent with previous findings [19,20], we identified two major TRM clusters in our dataset (Cluster 0 and 1) (Supplementary Figure S3A). Cluster 0 cells were similar to effector-like TRM cells, exhibiting higher expression of transcription factors including *Bhlhe40*, *Nr4a1*, *Nr4a2*, *Nr4a3*, *Rora*, *Klf2*, and *Klf3*. Conversely, Cluster 1 was similar to memory-like TRM cells, exhibiting high expression of *Id2*, *Id3*, *Jun*, *Fos*, *Cd160*, *Lag3*, and *Cxcr6* (Supplementary Figure S3B). Cluster 2 had a high level of mitochondrial gene expression, which may represent apoptotic cells (Supplementary Figure S3C,D). Cluster 3 had high expression of *S1pr1* and *Klf2*, but low expression of *Itgae* (encodes CD103) (Supplementary Figure S3C,D), indicating that Cluster 3 is likely composed of a small number of circulating memory cells contaminating the data.

**Figure 4:**
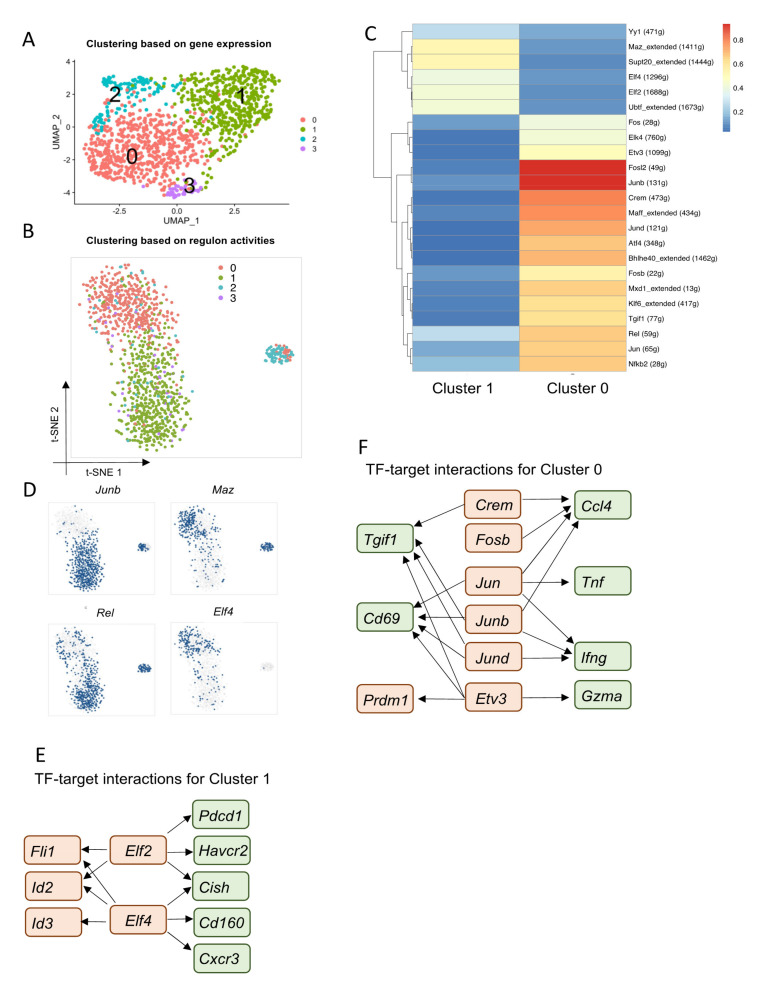
Core regulatory programs that determine heterogeneous TRM populations in siIELs: (**A**) Unsupervised clustering of TRM cells based on gene expression identified four major populations when visualized by UMAP. scRNA-seq data were generated from siIEL GP33^+^ CD44^+^ CD8^+^ cells, 30 days post-LCMV Armstrong infection. (**B**) Unsupervised clustering based on regulon activity separated cells into three major populations when visualized by tSNE. Colors denote the CD8^+^ T-cell subsets identified by gene expression profiles. (**C**) Heatmap showing the average regulon activity in Clusters 0 and 1. Scale bar denotes SCENIC AUC score. Numbers in parentheses denote the number of co-expressed and direct target genes of each transcription factor. (**D**) t-SNE projections showing binary regulon activity of example regulons for TRM subsets. (**E**,**F**) Gene regulatory networks showing TF-target interactions for Cluster 1 and Cluster 0. Key TFs are highlighted in red, putative regulated genes are highlighted in green.

Having validated that our TRM populations match those previously reported, we sought to investigate the core regulatory programs that determine the molecular and functional heterogeneity of the two TRM subsets. To do so, we performed SCENIC analysis on GP33^+^ CD44^+^ CD8^+^ T cells from siIELs 30 days post-LCMV-ARM infection. Unsupervised clustering based on regulon activities resulted in two major cell clusters, which were highly correlated with their gene expression profiles (Figure 4A,B). Cluster 1 had high activity of regulons such as *Yy1*, *Maz*, *Supt20*, *Elf2*, *Elf4*, and *Ubtf* (Figure 4C,D). Based on predictions of target genes of each TF from SCENIC analysis, we found that *Elf2* and *Elf4* might promote expression of inhibitory receptors (*Cd160*, *Pdcd1*, and *Havcr2* (encodes Tim3)), other TFs (*Fli1*, *Id2*, and *Id3*), *Cxcr3*, and *Cish* in Cluster 1 (Figure 4E). Cish is a member of the suppressor of cytokine signaling (SOCS) family, which has been shown to silence TCR signaling and suppress T-cell expansion, effector function, and cytokine polyfunctionality in CD8^+^ T cells [43]. Fli1 has been reported to function as a repressor of effector-cell differentiation during acute and chronic infection [38]. Therefore, *Elf2* and *Elf4* might be key TFs that regulate the quiescence of the memory-like TRM subset. Cluster 0 had high regulon activities of AP-1 TF family members, including *Jun*, *Junb*, *Jund*, *Fosb*, *Fosl2*, and *Fos* (Figure 4C,D). These were predicted by SCENIC to promote the expression of effector genes, such as *Tnf*, *Ifng*, and *Ccl4* in the effector-like TRM subset (Figure 4F). Furthermore, the activities of *Rel* and *Nfkb2*, which belong to the NF-κB signaling pathway, were found to be enriched in Cluster 0 (Figure 4C,D). We also found that *Elk4*, *Etv3*, *Crem*, *Maff*, *Atf4*, *Bhlhe40*, *Mxd1*, *Klf6*, and *Tgif1* were enriched in Cluster 0 (Figure 4C,D). By further checking the putative targets of these TFs, we found that *Etv3* might regulate effector function by promoting *Gzma* and *Prdm1* expression (Figure 4F). *Crem* has been reported to drive an inflammatory phenotype of T cells in patients with arthritis [44]. However, its role in regulating TRM heterogeneity is not clear. Together, these results revealed the gene regulatory networks that controlled the heterogeneity of TRM within the siIEL CD8^+^ T-cell pool.

## 4. Discussions

Functional heterogeneity within the memory CD8^+^ T-cell pool is critical for protection against various pathogens. Therefore, it is important to elucidate the key transcriptional regulators that control the differentiation and function of memory subsets. Our scRNA-seq and bulk RNA-seq analyses identified a number of known and unknown regulators. For example, our data suggested that *Tcf7* and *Lef1* regulated TCM formation; *Klf2*, *Klf3*, and *Nfatc3* may be critical for TEM and LLE subsets; and AP-1 TF family members and Nr4a TF family members may be required for TRM formation. Recent studies have begun to elucidate heterogeneity within the TRM cell pool. Our results identified several putative regulators, such as *Elf2* and *Junb*, that may control the formation of memory-like TRM and effector-like TRM, respectively. TRM cells from different tissues are known to exhibit similar features, and a core transcriptional profile of TRM cells has been identified: upregulation of tissue residency genes, inhibitory receptors, and TCR signaling molecules, and downregulation of genes controlling recirculation [45]. However, it has also been known that TRM cells are tissue-specific and may be regulated by distinct signals from different tissue microenvironments [33,46]. Here, we have studied the regulons of TRM cells from the small intestine, further studies are needed to identify the core regulators of TRM cells from other tissues.

Consistent with previous findings [20], we observed increased levels of transcripts associated with TCR signaling in TRM cells when compared with TCM and TEM cells, such as *Nr4a* TF family members and *Zap70*. These results suggested that TRM cells are experiencing TCR stimulation. The MAPK cascade is a critical mediator of T-cell receptor signals and has been shown to regulate the activation, effector function, and survival of CD8^+^ T cells [47,48,49]. Thus, the upregulation of MAPK signaling in TRM cells compared to TCM and TEM cells may correlate with the TCR sensitivity in TRM cells. Furthermore, AMPK is a critical regulator of lipid metabolism [50] and can maintain ATP levels in low glucose environments [51]. Given the importance of fatty acid metabolism to TRM cells, AMPK might regulate the metabolic fitness of TRM cells for them to survive in glucose-restricted but lipid-rich tissue environments.

Although phenotypic heterogeneity within the circulating memory CD8^+^ T-cell pool is well-characterized, the detailed gene regulatory networks among these subpopulations are not yet fully understood, especially for the newly identified LLE subset. CX_3_CR1 is a chemokine receptor for vascular endothelium-homing [29]. LLEs expressed the highest amount of CX_3_CR1, which may restrict them to circulation in the blood [52]. *Klrg1* and *Zeb2* are both related to terminally differentiated effector CD8^+^ T cells [7,30]. We found that *Tbx21*, *Elf4*, and *Nfatc3* are the major regulators of genes related to the migration patterns and terminally differentiated features of LLE cells. Furthermore, we found that TFs including *Etv3*, *Elk3*, and *Elf4* might regulate *Gzma* and *Gzmb* expression to promote the cytotoxicity of LLE cells. Short-lived effector T cells die by apoptosis after viral clearance, while LLE cells are a long-lived effector subset with low proliferative ability [3]. Therefore, further studies are needed to reveal the anti-apoptosis and cell survival regulators of LLE cells.

Gerlach et al. identified another subpopulation of circulating memory cells termed TPM, which is characterized by an intermediate level of CX_3_CR1 and is able to patrol between blood and peripheral tissues [2]. Here, this CX_3_CR1^int^ TPM is more equivalent to TEM in our scRNA-seq dataset. Our TEM subset exhibits intermediate levels of *Cx3cr1* and *Klrg1*. Furthermore, this TEM population expresses *Cxcr3*, which is known to guide T cells toward infected tissues [53], and *S1pr1*, which is known to promote T cell egress from SLOs and nonlymphoid tissues [27]. Therefore, the expression of *Cxcr3*, *S1pr1*, and intermediate level of *Cx3cr1* may be required for TEM cells to enter tissues and egress from the tissues back to the blood.

Recent studies have identified two major subsets of TRM CD8^+^ T cells during acute viral and bacterial infections, including effector-like Id3^lo^ Blimp1^hi^ TRM cells that are dominant in the early phase of infection and memory-like Id3^hi^B limp1^lo^ TRM cells that are prominent in the later phase of infections [19,20]. We also found two major TRM subsets in our dataset. Unfortunately, we did not have a good recovery of the *Prdm1* (encodes Blimp1) and *Id3* transcripts in our scRNA-seq data, and the expression levels of these two genes were comparable between these two major TRM populations (Supplementary Figure S3E). To validate the identity of our two TRM clusters, we integrated our scRNA-seq data with previously reported TRM subsets [19,20]. Unsupervised clustering showed overlap of our subsets with previously identified ones, indicating that our TRM Clusters 0 and 1 exhibited similar overall transcriptional profiles with effector-like Id3^lo^ Blimp1^hi^ TRM cells and memory-like Id3^hi^ Blimp1^lo^ TRM cells, respectively.

Our analyses also provide new insights regarding the distinct gene regulatory networks for the two TRM subsets. For example, *Elf2* and *Elf4* might regulate the quiescence of the memory-like TRM subset by promoting the expression of repressors for effector-cell differentiation, such as *Fli1* and *Cish*. On the other hand, the AP-1 TF family members may promote the expression of effector genes, such as *Tnf*, *Ifng*, and *Ccl4* in the effector-like TRM subset. Furthermore, we found that the effector-like TRM subset exhibited higher expression of NR4A TFs, suggesting that these cells undergo enhanced TCR signaling.

Overall, our work has generated a single-cell transcriptomic dataset of endogenous memory GP33^+^ CD8^+^ T cells from circulation and siIEL in response to acute LCMV infection. Our study identified a set of key regulators and core gene regulatory networks that program the migration patterns and functions of different circulating memory and TRM subsets. Our study not only demonstrates a systematic comparison of TRM cells to circulating memory T-cell populations but also may serve as a useful resource for studying the pathways that regulate the heterogeneity of TRM cells.

## Data Availability

The bulk RNA-seq and scRNA-seq data presented in this study are available in the GEO database with the accession code GSE181785.

## Supplementary Materials

The following are available online at https://www.mdpi.com/article/10.3390/cells10082143/s1, Figure S1: Sorting strategy for scRNA-seq, Figure S2: Differential analysis of gene expression profiles of TRM versus LLE and TEM, Figure S3: Single-cell transcriptomics identified heterogeneity of TRM from siIELs.
