# Supplementary material for: Single-Cell Transcriptomics Reveals Core Regulatory Programs That Determine the Heterogeneity of Circulating and Tissue-Resident Memory CD8+ T Cells
Source: Cells. 2021 Aug 20;10(8):2143. doi: 10.3390/cells10082143 (PMC8392357; doi:10.3390/cells10082143)
Supplement: Supplementary file 1 [file cells-10-02143-s001.zip › cells-1322418-supplementary.pdf]

cells

MDPI

Supplementary Material

Single-Cell Transcriptomics Reveals Core Regulatory Programs that Determine the Heterogeneity of Circulating and Tissue-Resident Memory CD8⁺ T Cells

Yao Chen [1,†], Jian Shen [1,2,†], Moujtaba Y. Kasmani [1,2], Paytsar Topchyan [1,2] and Weiguo Cui [1,2,*]

[1] Versiti Blood Research Institute, Milwaukee, WI 53213, USA; yaochen.immunity@gmail.com (Y.C.); jshen@mcw.edu (J.S.); mkasmani@mcw.edu (M.Y.K.); ptopchyan@mcw.edu (P.T.)
[2] Department of Microbiology and Immunology, Medical College of Wisconsin, Milwaukee, WI 53226, USA
* Correspondence: wcui@versiti.org
† These authors contributed equally.

A

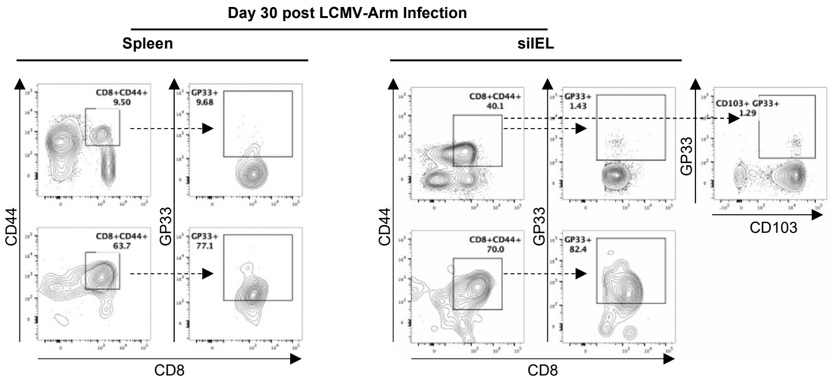

Figure S1. Sorting strategy for scRNA-seq: (**A**) Sorting strategy for CD8⁺ T cells from spleen (left) and small intestine (right).

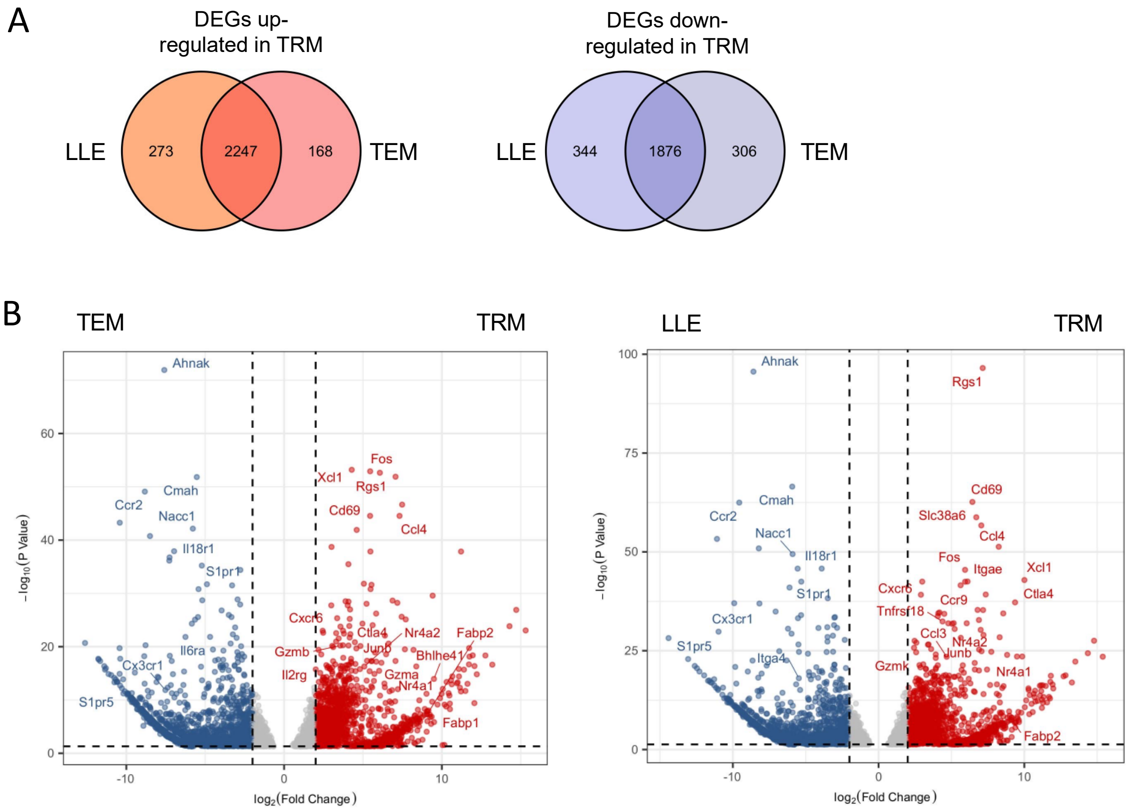

Figure S2. Differential analysis of gene expression profiles of TRM versus LLE and TEM. (**A**) Venn plots showing the overlaps of DEGs (p.adjust < 0.05) upregulated (red) or downregulated (blue) in TRM relative to LLE (left circle) and TEM (right circle). (**B**) Volcano plots showing the significantly (p.adjust < 0.05) differently expressed genes in each population.

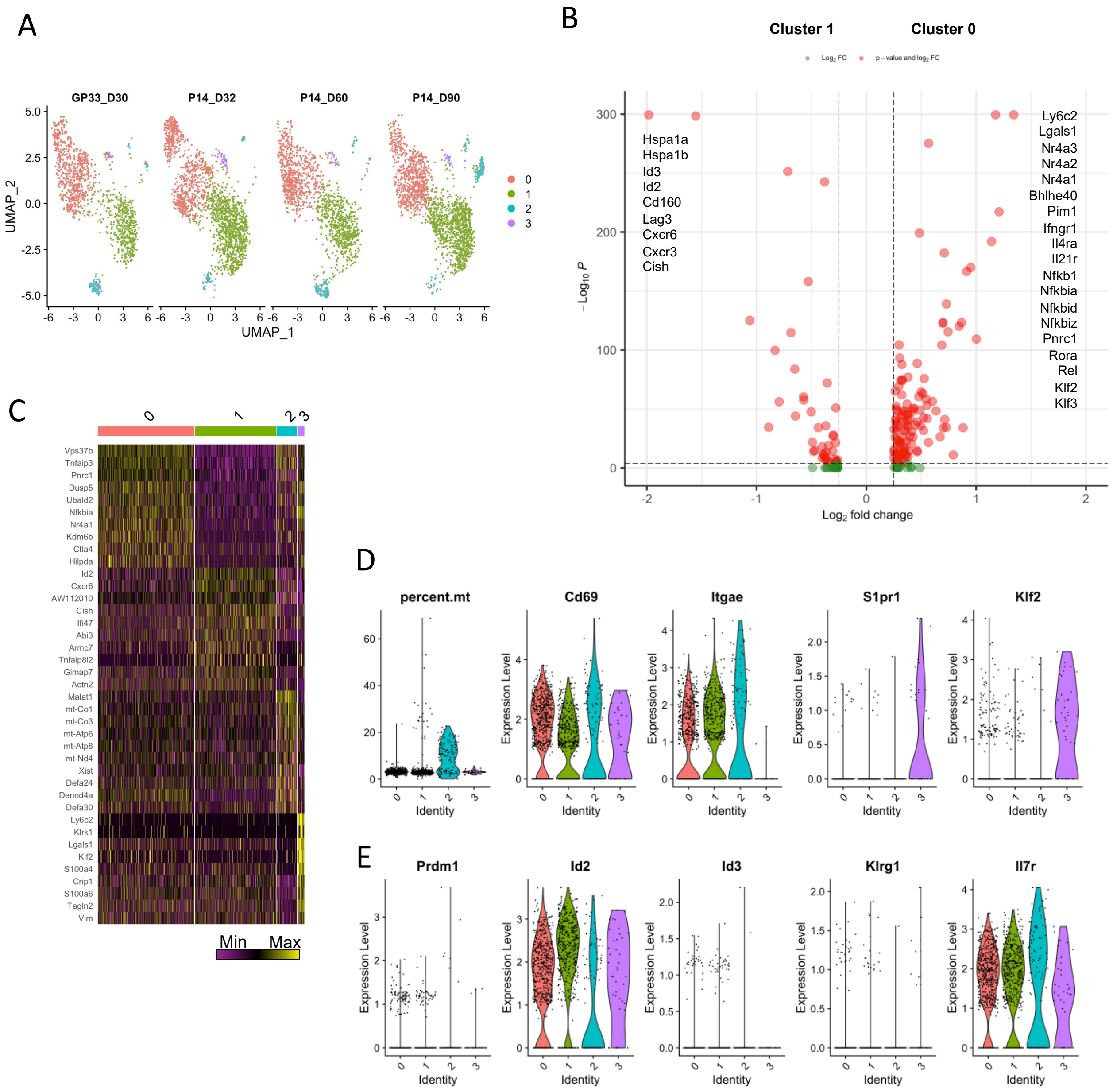

Figure S3. Single-cell transcriptomics identified heterogeneity of TRM from siIELs: (**A**) Unsupervised clustering based on gene expression identified four major TRM populations when visualized by UMAP. From left to right are scRNA-seq datasets generated from day 30 GP33⁺ cells, day 32 P14 cells, day 60 P14 cells, and day 90 P14 cells from siIELs. The P14 datasets were previously published (GSE131847). LCMV Armstrong infection was used in all datasets. Integration analysis was used to identify the shared cell types across different datasets. (**B**) Volcano plot showing genes differentially expressed between Cluster 0 and 1 TRM populations from scRNA-seq data. (**C**) Heatmap showing top 10 differentially expressed genes per cluster from the cells shown in (**A**). The color scale is based on a z-score distribution from—2 (purple) to 2 (yellow). (**D**–**E**) Violin plots showing the percentage of mitochondrial genes as well as the expression of signature TRM genes and subset-enriched genes.
